# Supplementary material for: AKT2 Loss Impairs BRAF-Mutant Melanoma Metastasis
Source: Cancers (Basel). 2023 Oct 12;15(20):4958. doi: 10.3390/cancers15204958 (PMC10605002; doi:10.3390/cancers15204958)
Supplement: Supplementary file 1 [file cancers-15-04958-s001.zip › cancers-2599871-supplementary.pdf]

Supplementary Materials:

AKT2 Loss Impairs BRAF-Mutant Melanoma Metastasis

Siobhan K. McRee[1,2], Abraham L. Bayer [3,4], Jodie Pietruska[2] , Philip N. Tsichlis[5], and Philip W. Hinds[1,2]

Listed:
Table S1: Primer Sequences and Hairpin Sequences
Table S2: Antibodies Used
Figure S1:. Characterization of AKT Phosphorylation and Knockdown Cell Lines
Figure S2: AKT2 knockdown impairs wound healing, migration and invasion in WM455 and UACC903 cells.
Figure S3: Activation state of AKT isoforms in murine melanoma cells *in vitro* and *in vivo*.
Figure S4: AKT1 Deletion Delays Primary Melanoma Growth and Improves Survival
Figure S5: AKT1 Knockdown impairs Melanoma Cell Proliferation
Figure S6: AKT1 Knockdown Restricts Anchorage Independent Growth

qPCR Target	Forward	Reverse
ECAD	GAACGCATTGCCACATACAC	GAATTCGGGCTTGTTGTCAT
ZEB1	GCACCTGAAGAGGACCAGAG	TGCATCTGGTGTTCCATTTT
MMP2	CCGTCGCCCATCATCAAGTT	CTGTCTGGGGCAGTCCAAAG
Snail	CACTATGCCGCGCTCTTT	GGTCGTAGGGCTGCTGGAA
TEAD2	CTCACTCCGTAGAAGCCACC	TGCCTTCTTCCTGGTCAAGT
TEAD3	GCACCTTCTTCCGAGCTAGA	TACGGCCGAATGAGTTGATT
TBP	GAGCCAAGAGTGAAGAACAGTC	GCTCCCCACCATATTCTGAATCT
**shRNA Hairpins**		
AKT1	CCGGGAGTTTGAGTACCTGAAGCTGCTCGAGCAGCTTCAGGTACTCAAACTCTTTTTG	
AKT2	CCGGGCGTGGTGAATACATCAAGACCTCGAGGTCTTGATGTATTCACCACGCTTTTTG	
AKT3	CCGGCTGCCTTGGACTATCTACATTCTCGAGAATGTAGATAGTCCAAGGCAGTTTTTG	
Non-Targeting	CCGGCAACAAGATGAAGAGCACCAACTCGAGTTGGTGCTCTTCATCTTGTTGTTTTT	

Table S1: Primer Sequences and Hairpin Sequences

Antibody	Source	Catalog Number
AKT1	Cell Signaling Technologies	2938
AKT2	Cell Signaling Technologies	5239
AKT3	Cell Signaling Technologies	8018
Alpha Tubulin	Cell Signaling Technologies	3873
Beta Actin	Sigma	A5441
p-RXRXXS/T	Cell Signaling Technologies	9611
pan AKT	Cell Signaling Technologies	4691
pan phospho-AKT	Cell Signaling Technologies	4060
PDHE1a	Cell Signaling Technologies	3205
PDHK1	Cell Signaling Technologies	3820
phospho-AKT1	Cell Signaling Technologies	9018
phospho-AKT2	Cell Signaling Technologies	8599
phospho-PDHE1a	Cell Signaling Technologies	31866
PTEN	Cell Signaling Technologies	9552

Table S2: Antibodies Used

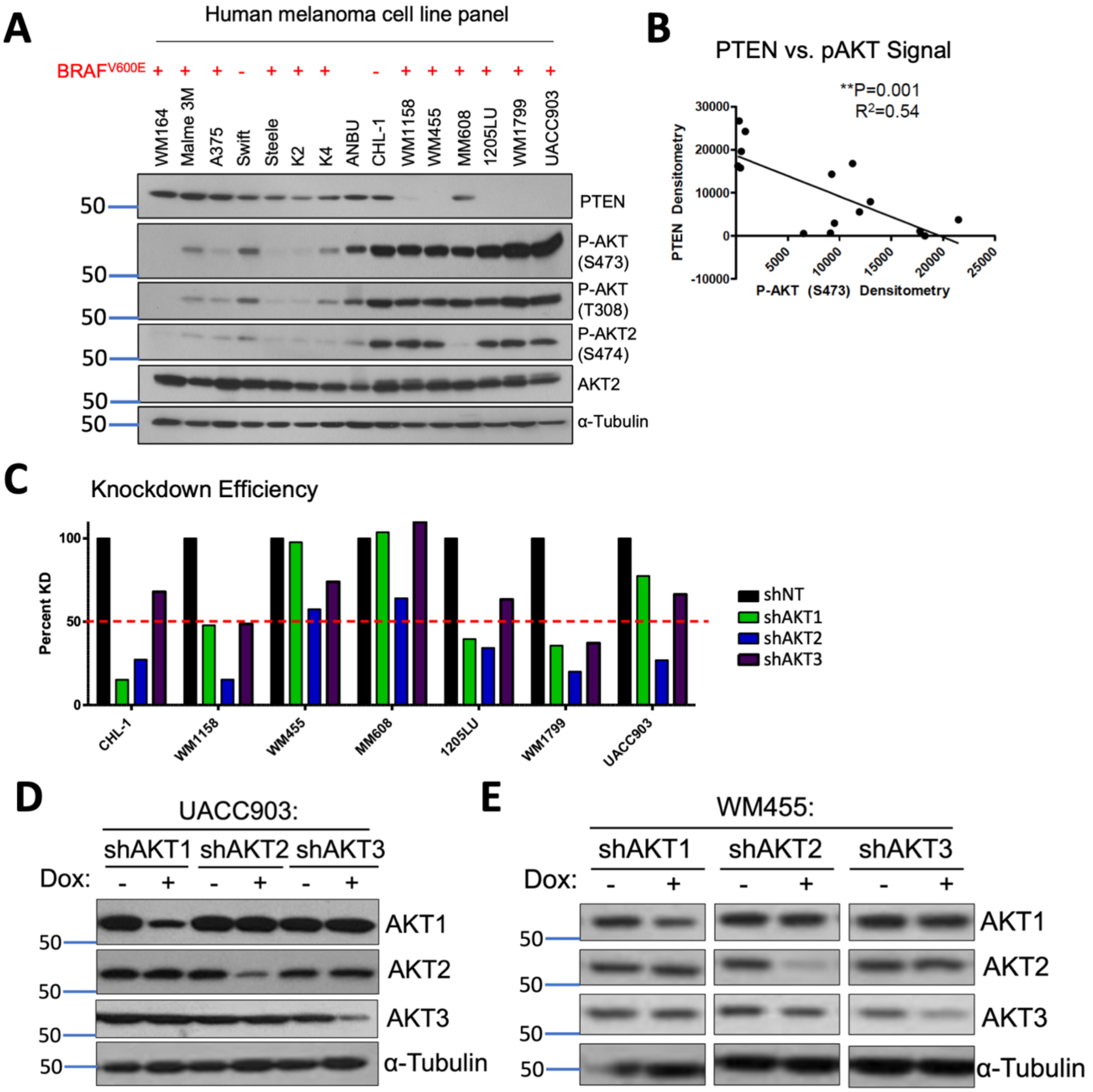

Supplementary Figure S1. Characterization of AKT Phosphorylation and Knockdown Cell Lines A. Immunoblotting for PTEN, AKT phosphorylation (T308, S473) and AKT2 protein and phosphorylation (S474) phosphorylation in a panel of human melanoma cell lines. A representative image is shown from at least two independent experiments, with alpha-tubulin used as a loading control. B. Analysis of densitometry of immunoblotting comparing total AKT phosphorylation (S473) to PTEN protein level. C. Akt-isoform knockdown efficiency was quantified from western blots using ImageJ in novel generated human melanoma cell lines after 72h of DOX treatment (0.5ug/mL) and normalized to DMSO-treated and loading controls. D-E. Representative immunoblots of human melanoma cell lines UACC903 (D) and WM455 (E) showing Akt-isoform knockdown efficiency after 72h DMSO or doxycycline exposure (0.5ug/mL).

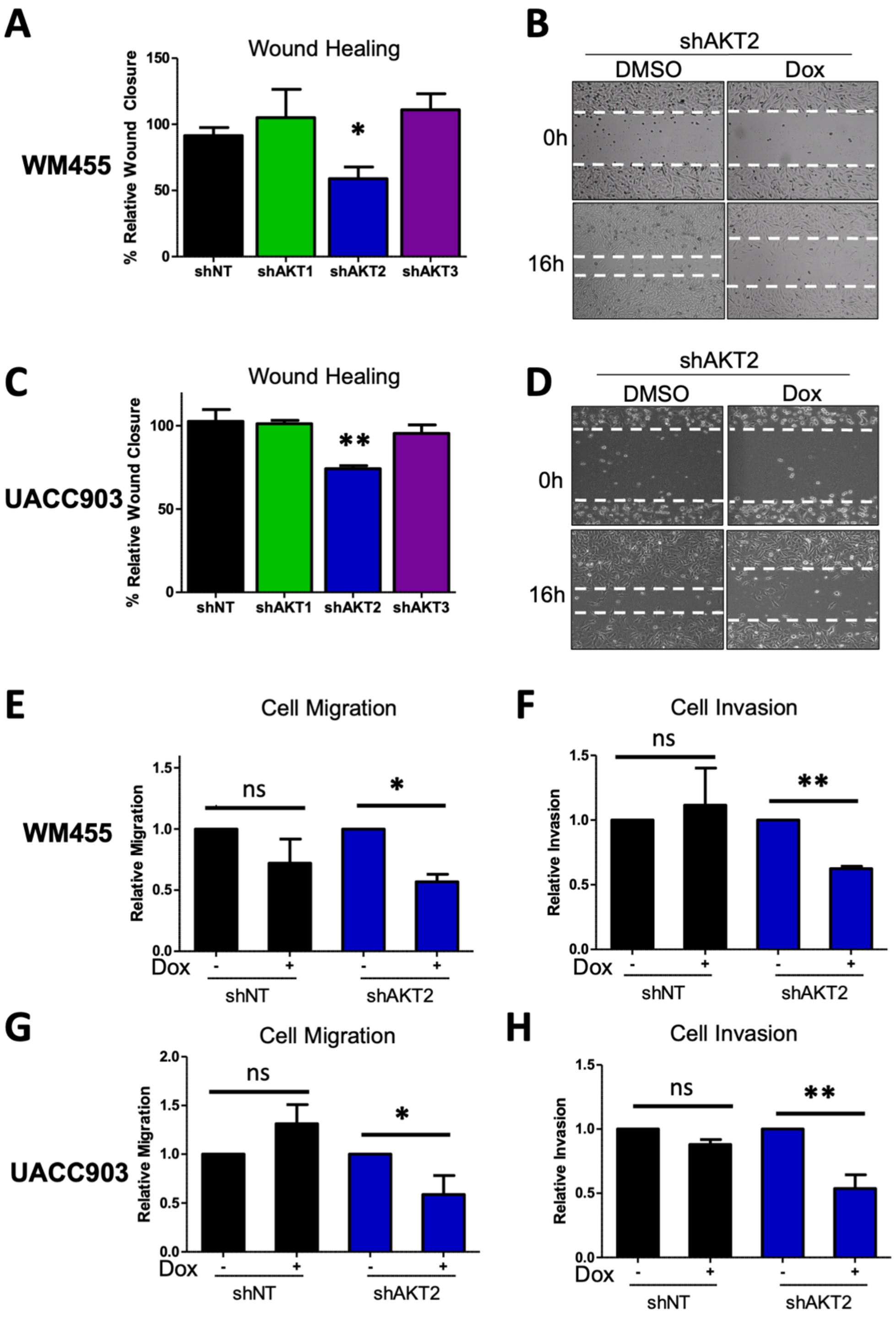

Supplementary Figure S2. AKT2 knockdown impairs wound healing, migration and invasion in WM455 and UACC903 cells.

A-D. Wound closure in indicated cell lines at 0h and 16h post scratch in AKT2 KD cells treated with DMSO- or doxycycline (DOX)-containing media. Shown are quantification from 3 independent experiments for DOX relative to DMSO treated WM455 cells (**A**) or UACC903 cells (**C**), with representative images at 4x magnification of control (NT) or isoform-specific KD cells

treated with DOX or DMSO at 0 and 16 hours for WM455 cells (**B**) or UACC903 cells **(D).** Cell migration ability after treatment in DMSO or DOX containing media using transwell assay for WM455 cells (**E**) or UACC903 cells (**G**) from 3 independent experiments. Cell invasion through Matrigel coated membranes after treatment in DMSO or DOX containing media for WM455 cells (**F**) or UACC903 cells (**H**) from 3 independent experiments.

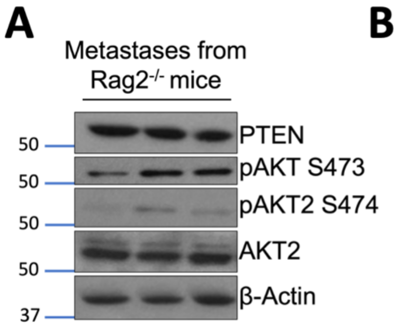

B

Cell Line	$BRAF^{V600E}$	INK4A	ARF	Notes
SM1	+	WT	WT	Generous gift from Toni Ribas
SM1-750	+	WT	WT	SM1 cells passaged in BL6 mice and infected with pLENTI-luciferase
TUMM4218	+	-/-	-/-	
TUMM4228	+	WT	-/-	
TUMM4246	+	WT	-/-	
TUMM8253	+	WT	-/-	Mouse received UV radiation at PND 3.5

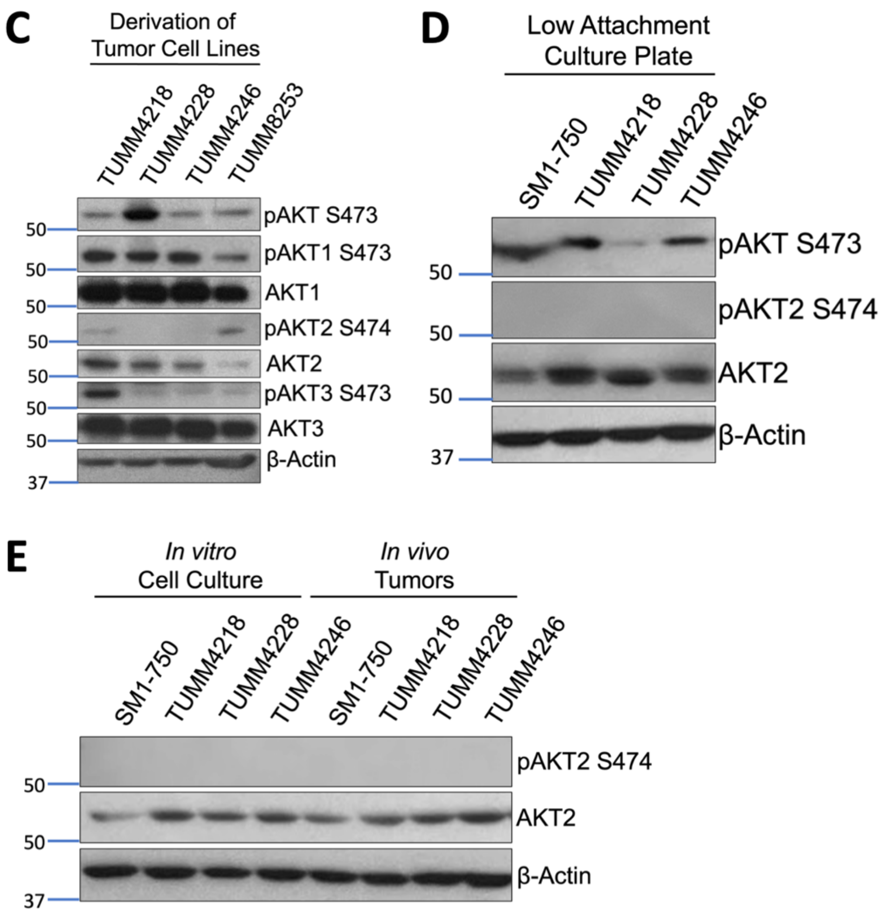

Supplementary Figure S3. Activation state of AKT isoforms in murine melanoma cells *in vitro* and *in vivo*. A. SM1-750 cells were injected into the tail vein of Rag2-/- mice and metastatic nodules were collected after 4 weeks, or when mice became moribund, homogenized and subjected to immunoblotting. **B.** Table characterizing SM1 and SM1-750 derived cells (Figure 3) and lines derived form spontaneous primary melanomas in mice expressing human BRAFV600E transgene after isolation and passage in culture. TUMM: Tufts University Mouse Melanoma. PND: post-natal day **C.** Expression and phosphorylation of each AKT isoform was characterized by immunoblotting. **D.** SM1-750 and TUMM cell lines were cultured in low-attachment plates until macroscopic colonies were visible, then collected and subjected to

immunoblotting. **E.** SM1-750 and TUMM cell lines were grown in standard 2-D adherent culture, or injected subcutaneously into NODSCID mice and the resulting tumors collected and subjected to immunoblotting with indicated antibodies.

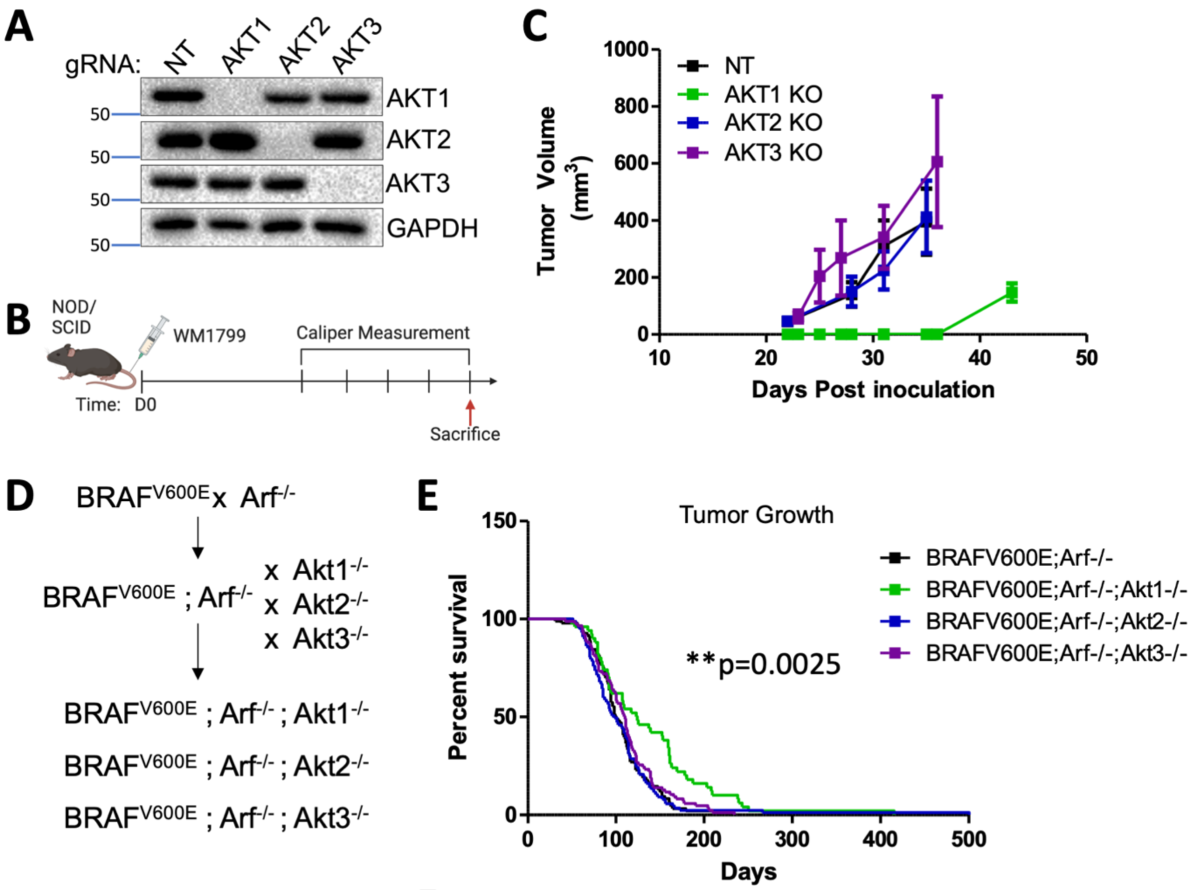

F

Genotype	Median Survival (Days)	P-Value	Cohort Size (#)	Tumor Incidence
BRAF^{V600E}; Arf$^{-/-}$	102	--	96	54/96 (56.3%)
BRAF^{V600E}; Arf$^{-/-}$; Akt1$^{-/-}$	125	**0.0025	50	27/50 (54%)
BRAF^{V600E}; Arf$^{-/-}$; Akt2$^{-/-}$	101	n.s.	87	45/87 (51.7%)
BRAF^{V600E}; Arf$^{-/-}$; Akt3$^{-/-}$	109.5	n.s.	86	45/86 (52.3%)

Supplementary Figure S4. AKT1 Deletion Delays Primary Melanoma Growth and Improves Survival. A. Representative immunoblot for isoform specific CRISPR KO WM1799 cells. **B.** Experimental schematic in which NT or AKT isoform specific WM1799 cells were injected into the flank of NOD/SCID mice and tumor growth was monitored by Caliper measurement over time, quantified in **C** from n=5-10 mice / group**. D.** Breeding scheme for creation of melanoma prone Akt isoform KO mice by cross with BRAF^{V600E}ARF^{-}/- mice. **E.** Survival curve representing percent overall survival of AKT isoform KO melanoma prone mice over time, with summarized data and cohort numbers in **F.**

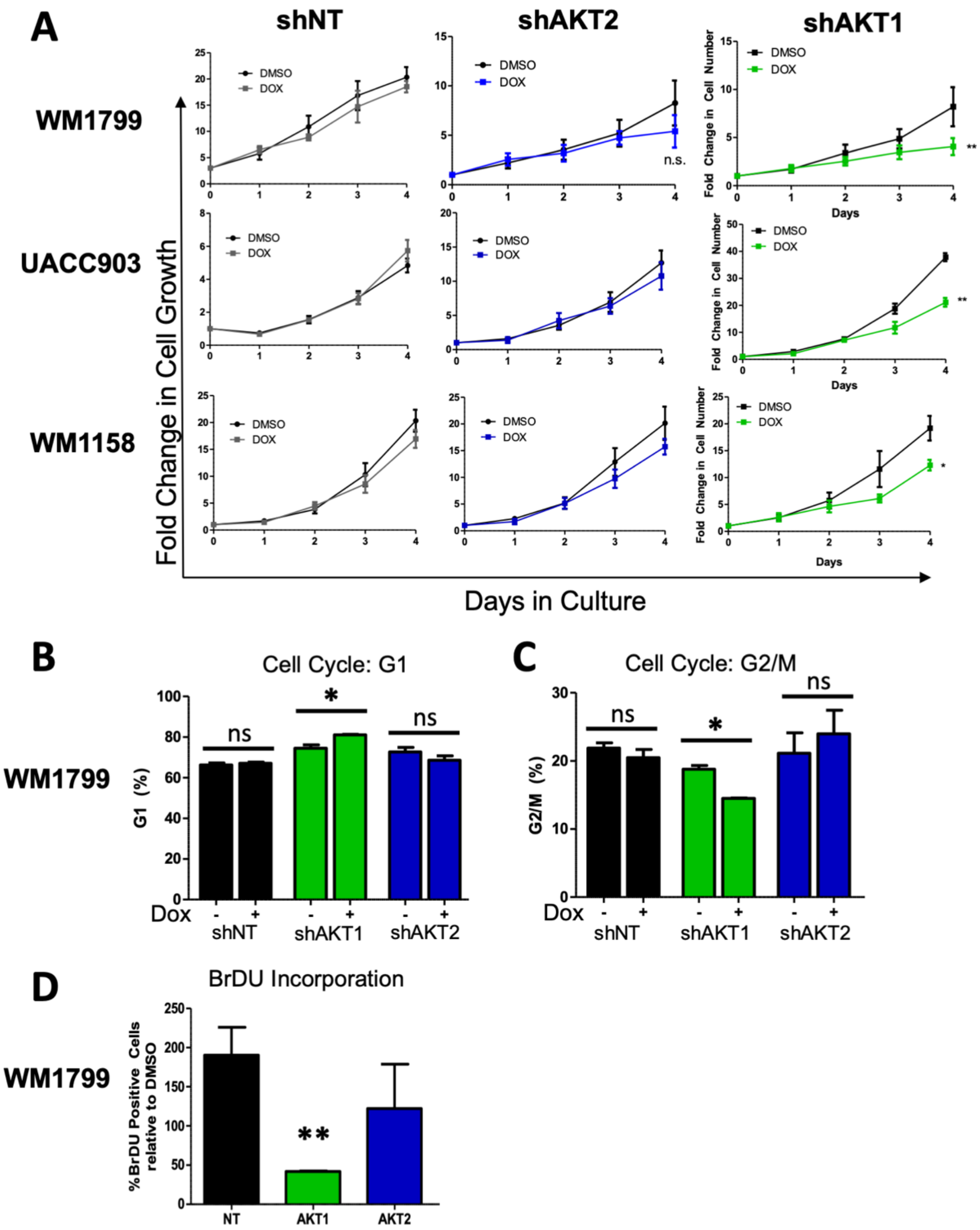

Supplementary Figure S5. AKT1 Knockdown impairs Melanoma Cell Proliferation. A. Cell pr oliferation of human melanoma cell lines expressing non-targeting (shNT), AKT2 (shAKT2), or AKT1 (shAKT1) hairpins in the presence of DMSO- or DOX-containing media assessed using cell counting with trypan blue exclusion and represented as a fold change from day 0 over 4 days, from 3 independent experiments. **B-C.** Cell cycle analysis by propidium iodide staining to assess G1 or G2/M fraction of WM1799 cells expressing shNT or shAKT isoform hairpins in the presence (+) or absence (-) of DOX-containing media from 3 independent experiments. **D.** BrDu incorporation was quantified from WM1799 cells expressing inducible AKT-isoform or NT hairpins and grown in the presence of DOX or DMSO for 2 days before adding BrDu for one hour, plotted relative to DMSO treated cells from 3 independent experiments.

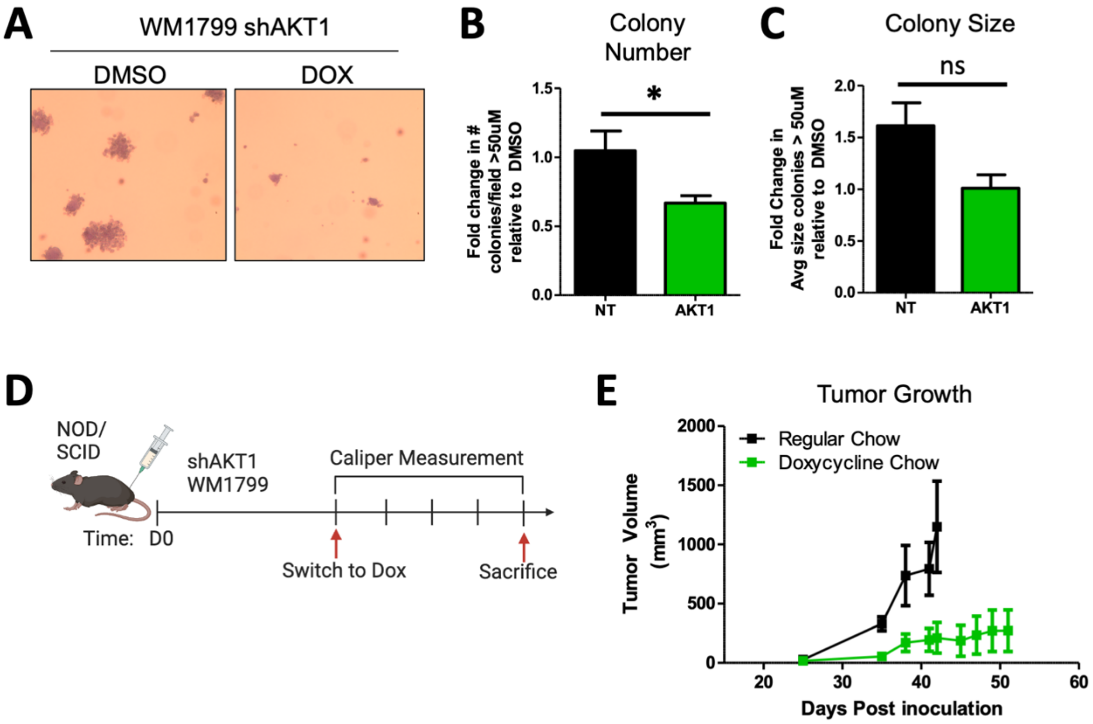

Supplementary Figure S6. AKT1 Knockdown Restricts Anchorage Independent Growth
A. Anchorage-independent growth of WM1799 shAKT1 cells in soft-agar and incubated with DMSO or DOX-containing media. Colonies were fixed and stained with crystal violet and colony number **(B)** or colony size **(C)** greater than 50uM were counted using ImageJ from 3 independent experiments. **D.** Experimental scheme showing WM1799 shAKT1 cells were injected subcutaneously into NOD/SCID mice and maintained on either regular or DOX containing chow (n=3-4 mice / group). Mice were sacrificed when tumors reached $1500m^3$ according to approved protocols. **E.** Tumor growth by caliper measurement was quantified over time.
